# Treatment Outcome of Acute Promyelocytic Leukemia with Modified Aida Protocol

**DOI:** 10.1155/2010/672137

**Published:** 2010-05-16

**Authors:** Kátia B. Barbosa Pagnano, Gustavo de Carvalho Duarte, Irene Lorand-Metze, Márcia Torresan Delamain, Eliana Cristina Miranda, Cármino Antonio De Souza

**Affiliations:** Hematology and Hemotherapy Center, University of Campinas, SP, Rua Carlos Chagas 480, Campinas 13083-970, Brazil

## Abstract

We analyzed the outcome of a series of 19 newly diagnosed patients with acute promyelocytic leukemia treated with AIDA modified protocol, using mitoxantrone in place of idarubicin. Eleven patients achieved morphologic CR (58%). The remaining 8 patients had induction failure due to death during induction. Ten of eleven patients in CR achieved molecular remission after induction therapy and all the 8 patients had molecular remission after consolidation. Eight patients completed the three consolidation courses as scheduled and then proceeded to maintenance therapy. After a median follow up of 52 months, no molecular or hematological relapse has occurred. The 4-year disease-free survival is 82%. The study showed the antileukemic efficacy of mitoxantrone and that it could be used as a reasonable option in anthracycline-based strategies in APL.

The simultaneous administration of all-*trans*retinoic acid (ATRA) and anthracycline-based chemotherapy is currently considered the standard induction therapy in newly diagnosed patients with acute promyelocytic leukemia (APL), leading to complete remission (CR) rate greater than 90% and potential cure in up to 80% [1, 2]. Once in CR, the standard postremission therapy consists of 2-3 cycles of anthracycline-based chemotherapy followed by ATRA-containing maintenance therapy [3]. Most common anthracyclines used are idarubicin or daunorubicin. The aim of this study was to evaluate the clinical outcome of APL patients treated with a modified AIDA protocol [4] in which idarubicin was replaced by mitoxantrone at an equivalent dose (1 mg of idarubicin = 1 mg of mitoxantrone). This replacement was made due to the economical difficulty of buying idarubicin in our center.

We analyzed the outcome of a series of newly diagnosed patients with APL treated at the Hematology Center of the University of Campinas. Diagnosis was confirmed by the presence of t(15; 17) in cytogenetic studies and/or PML/RAR*α* gene rearrangement [5, 6]. Induction chemotherapy consisted of intravenous mitoxantrone (10 mg/m^2^) on days 2, 4, 6, and 8 and oral ATRA from day 1 (45 mg/m^2^/d) until complete remission. Patients in CR received three cycles of consolidation therapy: cycle one: mitoxantrone 5 mg/m^2^ and cytarabine 1 g/m^2^ IV(days 1–4); cycle 2: mitoxantrone 10 mg/m^2^ and vepeside 100 mg/m^2^ IV (days 1–5); cycle 3: mitoxantrone 10 mg/m^2^, cytarabine 150 mg/m^2^/8 h IV (days 1–5), and tioguanine 70 mg/m^2^/8 h (days 1–5). Maintenance therapy consisted of oral ATRA (45 mg/m^2^/d) during 14 days every 3 months for 2 years. Actuarial survival curves were calculated by the Kaplan-Meier method. 

Between March 1999 and May 2006, 19 patients with APL were treated with the previously described AIDA modified protocol with mitoxantrone replacing idarubicin. The main clinical and biologic characteristics of the 19 patients are described in Table 1. Eleven patients achieved morphologic CR (58%). The remaining 8 patients had induction failure due to death during induction, 4 attributable to cerebral or pulmonary hemorrhage (50%), 3 to infection, and one to differentiation syndrome. Eight deaths occurred among the 10 patients with white blood cell count (WBC) at presentation greater than 2.7 × 10^9^/L (median), while the remaining 3 deaths occurred among 9 patients with WBC less than 2.7 × 10^9^/L (*P* = .01). Ten of 11 patients who achieved CR proceeded to consolidation therapy. One patient died from pulmonary *Mycobacterium tuberculosis* infection before consolidation. Two of 10 patients died during consolidation, one after the first cycle and one after the third cycle, both due to infection. The remaining 8 patients completed the three consolidation courses as scheduled, and then proceeded to maintenance therapy. One patient interrupted maintenance therapy due to a second episode of pancreatitis. Ten of eleven evaluable patients achieved molecular remission after induction therapy, and all the 8 patients had molecular remission after consolidation. After a median follow-up of 52 months, no molecular or hematological relapse has occurred. The 4-year disease-free survival is 82% (Figure 1) and the cumulative incidence of relapse was 0% in this population.

 The problem of the high mortality rate during induction was recently addressed in a retrospective study of Brazilian APL patients treated in different centers [7] and probably was related to a deficient supportive therapy. Apart from confirming the high mortality during induction and consolidation, our study provides other data that we consider of interest to be reported. 

Despite the small sample size, the present study shows the antileukemic efficacy of mitoxantrone in APL, leading to a high rate of molecular remission after induction therapy and the lack of relapses in those patients who completed induction and consolidation therapy. This study suggests that mitoxantrone could be considered as an alternative to other anthracyclines in this disease if these drugs are not available.

## Figures and Tables

**Figure 1:**
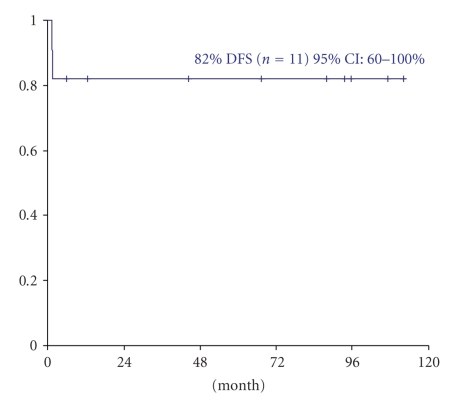
Disease-free survival of APL patients.

**Table 1:** Demographic and baseline characteristics of the study population.

Characteristic	Total	
	Median (range)	No. (%)
No. of patients		19 (100)
Age, year	40 (14–68)	
15 or younger		1 (5)
16–60		17 (90)
61 or older		1 (5)
Gender		
Male		10 (53)
Female		9 (47)
WBC count, × 10^9^/L	2.7 (0.4–49.0)	
Less than 5		10 (52)
5–10		3 (16)
11–50		6 (32)
PB blast count, ×10^9^/L	60 (1–98)	
Less than 30		3 (16)
30 or higher		16 (84)
Platelet count, ×10^9^/L	13 (4–85)	
Less than 40		15 (79)
40 or higher		4 (21)
Hemoglobin, g/dL	8.8 (5.0–10.6)	
Less than 10		17 (90)
10 or higher		2 (10)
Creatinine, mg/dL	0.92 (0.59–1.47)	
Less than 1.4		18 (95)
1.4 or higher		1 (5)
Coagulopathy		
No		7 (37)
Yes		12 (63)
Morphologic subtype		
Hypergranular		15 (79)
Microgranular		4 (21)
Relapse risk group		
Low		3 (16)
Intermediate		11 (58)
High		5 (26)
PML/RAR*α* isoform		
BCR1/BCR2		10 (59)
BCR3		7 (41)
Not available		2
